# Comparison of gene expression profiling between lung fibrotic and emphysematous tissues sampled from patients with combined pulmonary fibrosis and emphysema

**DOI:** 10.1186/1755-1536-5-17

**Published:** 2012-10-01

**Authors:** Masayuki Hanaoka, Michiko Ito, Yunden Droma, Atsuhito Ushiki, Yoshiaki Kitaguchi, Masanori Yasuo, Keishi Kubo

**Affiliations:** 1 First Department of Medicine, Shinshu University School of Medicine, 3-1-1 Asahi, Matsumoto, Japan

**Keywords:** Emphysematous lesion, Cellular fraction, Fibrotic lesion, Gene expression profiles, Immune system, Lung

## Abstract

**Background:**

Combined pulmonary fibrosis and emphysema (CPFE) is characterized by both emphysema of the upper zone and diffuse parenchymal lung disease with fibrosis of the lower zone of the lung on chest computed tomography. The aim of this study was to investigate the mechanism of CPFE regarding gene expressions by comparing the results of microarray sequences between fibrotic and emphysematous lesions in the lungs of CPFE patients.

**Results:**

The expression profiles of the fibrotic and emphysematous lesions were remarkably different in terms of function. Genes related to the immune system, structural constituents of the cytoskeleton, and cellular adhesion were overexpressed in fibrotic lesions, while genes associated with the cellular fraction, cell membrane structures, vascular growth and biology, second-messenger-mediated signaling, and lung development (all processes that contribute to the destruction and repair of cells, vessels, and the lung) were overexpressed in emphysematous lesions.

**Conclusions:**

The differences in gene expression were detected in fibrotic and emphysematous lesions in CPFE patients. We propose that the development of coexisting fibrotic and emphysematous lesions in CPFE is implemented by these different patterns of gene expressions.

## Background

One of the most demonstrably clinical features of combined pulmonary fibrosis and emphysema is that emphysema of the upper zones and diffuse parenchymal lung disease with fibrosis of the lower zones of the lungs are both presented on chest computed tomography [1]. The distinctive features of CPFE include cigarette smoking, severe dyspnea, hypoxemia at exercise, subnormal spirometry findings, and severely impaired lung diffusion capacity [1,2]. Severe pulmonary hypertension is frequently observed in CPFE, and is thought to determine its prognosis [1,3]. The substantial pathogenesis of CPFE is still unresolved because CPFE is not just one identical phenotype of either idiopathic pulmonary fibrosis (IPF) or emphysema [1,2]. Genetic factors have been shown to play significant roles in the development of IPF. Fibrotic lung specimens from patients with IPF exhibited misexpressions of genes encoding proteins probably involved in the metabolism of the extracellular matrix (ECM), chemokines, and tissue remodeling [4]. Genetic factors are also believed to be associated with the pathogenesis of emphysema. Lung tissues from patients with severe emphysema displayed irregular expression of genes involved in inflammation, the ECM, cytokines, chemokines, apoptosis, and stress responses [5].

We hypothesized that coexistent fibrosis and emphysema are programmed by differential gene expressions in the corresponding lesions in the lungs of smokers susceptible to CPFE. Given the importance of genetic susceptibility in understanding the etiology and pathogenesis of CPFE, we performed the whole-genome microarray to sequence the gene expression profiling on fibrotic and emphysematous tissues sampled from three patients with CPFE to identify genes distinguishably expressed in fibrotic lesions and emphysematous lesions in lung tissues of patients with CPFE.

## Results

### Data deposition

The data reported in this paper have been deposited in the Gene Expression Omnibus database [GEO:GSE38934].

**Figure 1:**
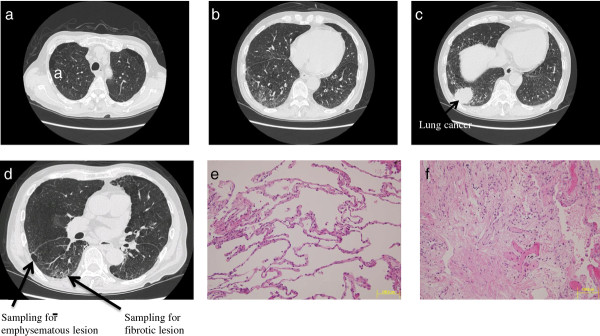
**Chest computed tomography and H & E staining microscopic images for case 1.** Upper panel: emphysematous change in the upper zone of the lung (**a**), fibrotic lesion at the lung base (**b**), and the cancer shadow (arrow) at the right inferior lobe (**c**). Lower panel: positions (arrows) of sampling in the emphysematous lesion and the fibrotic lesion in the right inferior lobe without cancerous lesions (**d**), and the H & E staining microscopic images of specimens of the emphysematous lesion (**e**) and the fibrotic lesion (**f**).

**Figure 2:**
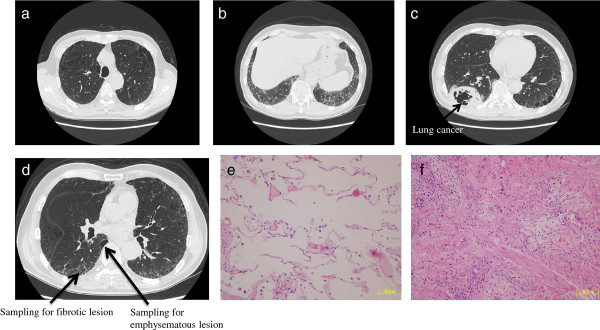
**Chest computed tomography and H & E staining microscopic images for case 2.** Upper panel: emphysematous change in the upper zone of the lung (**a**), fibrotic lesion at the lung base (**b**), and the cancer shadow at the right inferior lobe (**c**). Lower panel: positions (arrows) of sampling in the emphysematous lesion and the fibrotic lesion in the right inferior lobe without cancerous lesions (**d**), and the H & E staining microscopic images of specimens of the emphysematous lesion (**e**) and the fibrotic lesion (**f**).

**Figure 3:**
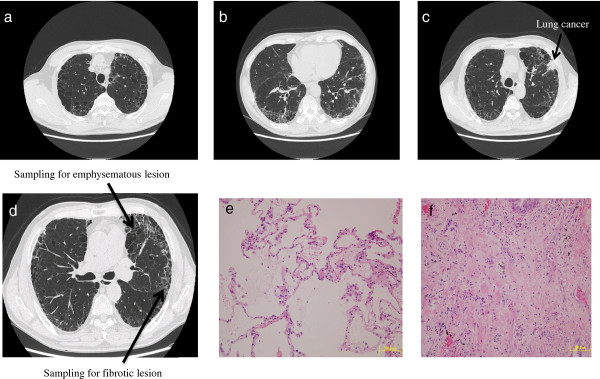
**Chest computed tomography and H & E staining microscopic images for case 3.** Upper panel: emphysematous change in the upper zone of the lung (**a**), fibrotic lesion at the lung base (**b**), and the cancer shadow at the left superior lobe (**c**). Lower panel: positions (arrows) of sampling in the emphysematous lesion and the fibrotic lesion in the left superior lobe without cancerous lesions (**d**), and the H & E staining microscopic images of specimens of the emphysematous lesion (**e**) and the fibrotic lesion (**f**).

Among all the specimens of lung tissue from the resected lobes of the six patients (see Methods), six lung specimens from three patients with CPFE (three specimens from fibrotic lesions and three specimens from emphysematous lesions) were selected to apply to the current gene expression profiling study after confirming the pathology in fibrotic lesions and emphysematous lesions by H & E staining. Figures 1, 2, 3 show the chest computed tomography and H & E staining microscopic images for each case, representatively showing the upper zone with emphysema, the lower zone with fibrosis, the cancer shadow, and the position of sampling without cancerous lesions. All three patients were male ex-smokers (smoking from 38 to 72 pack-years) aged from 60 to 78 years. The specimens of fibrotic lesions showed usual interstitial pneumonia in histology, characterized by fibroblastic foci and excessive deposition of the ECM (Figures 1f, 2f, 3f).

### Summary of *t* test results, volcano plot and hierarchical clustering analysis

**Figure 4:**
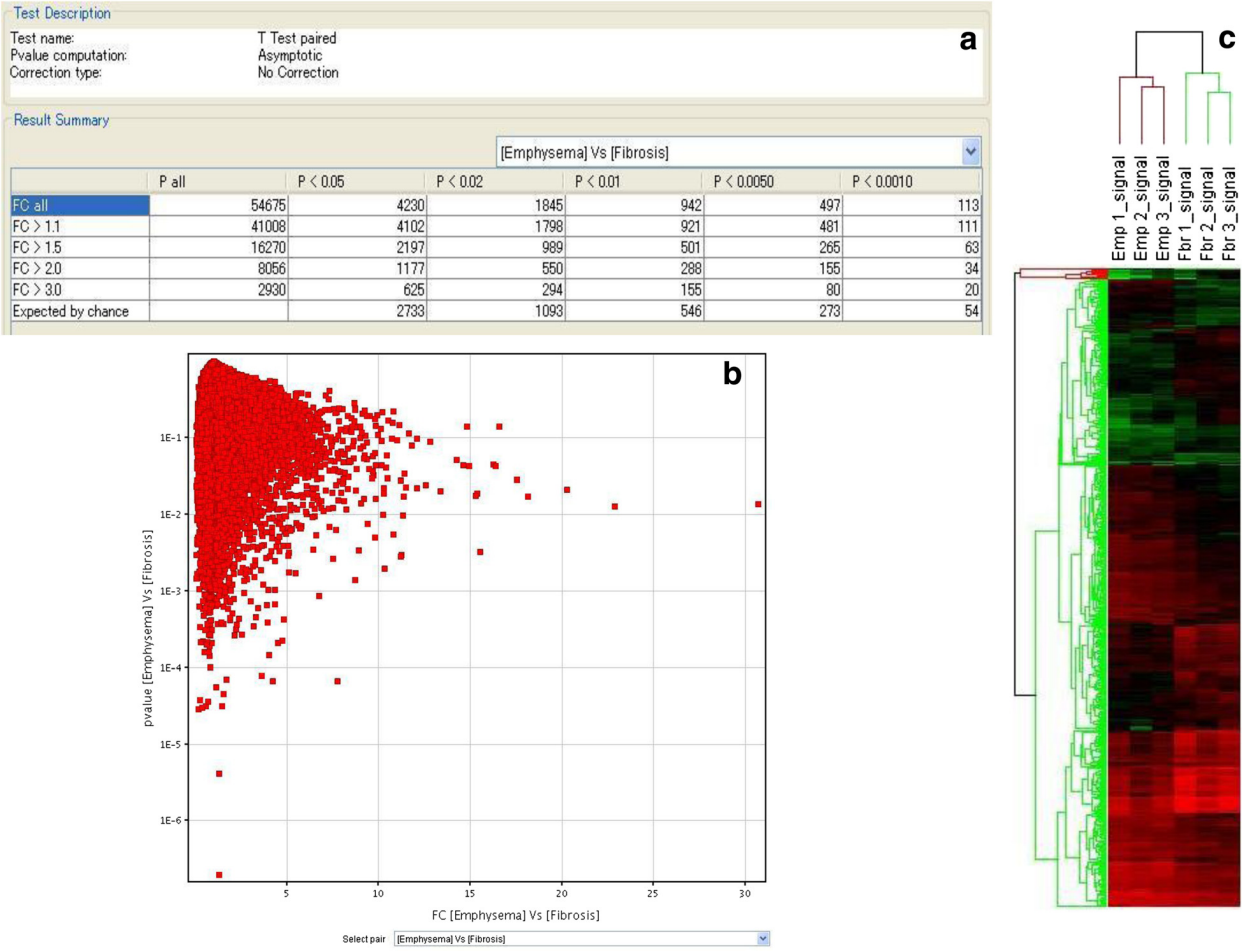
**Comparison of the signals between emphysematous and fibrotic lesions, fold-change and*P*value intersection, and hierarchical clustering analysis.** Paired *t* test results summary comparing between emphysematous and fibrotic lesions on the microarray data (**a**), volcano plot representing the point of intersection of fold-change (FC) (emphysema vs. fibrosis) relative to *P* value (emphysema vs. fibrosis) (**b**), and heat map built as the result of hierarchical clustering analysis (**c**). Emp 1_singal, Emp 2_singal, and Emp 3_singal indicate the signals detected in the specimens of emphysematous lesions in case 1, case 2, and case 3, respectively. Fbr 1_singal, Fbr 2_singal, and Fbr 3_singal indicate the signals detected in the specimens of fibrotic lesions in case 1, case 2, and case 3, respectively.

Figure 4 presents the paired *t* test results summary as a comparison between fibrotic and emphysematous lesions on the microarray data (Figure 4a) and the volcano plot as a representation of the point of intersection of the fold-change (emphysema vs. fibrosis) relative to the *P* value (emphysema vs. fibrosis) (Figure 4b). Hierarchical clustering analysis was performed to build a heat map (Figure 4c), which beautifully separated fibrotic and emphysematous lesions into two clusters.

### Gene functional classification for fibrotic lesions in CPFE

**Figure 5:**
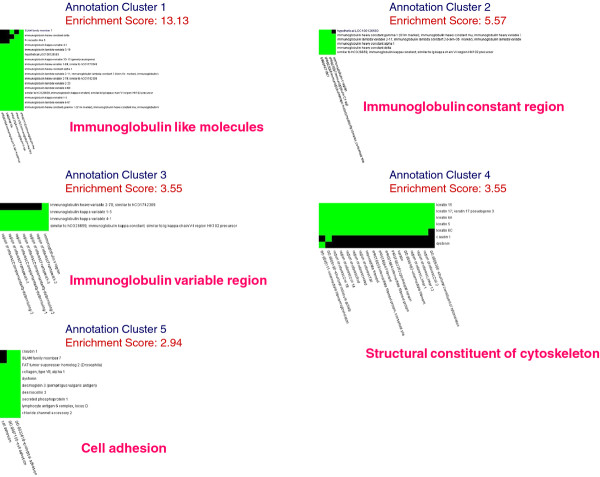
**Highly related genes and corresponding annotations in fibrotic lesions of combined pulmonary fibrosis and emphysema.** Two-dimensional view maps of the relationships between highly related genes and their corresponding annotations in fibrotic lesions of combined pulmonary fibrosis and emphysema (CPFE) using the Gene Functional Classification Tool ( http://david.abcc.ncifcrf.gov/gene2gene.jsp) from the Database for Annotation, Visualization and Integrated Discovery (DAVID; http://david.abcc.ncifcrf.gov/home.jsp). Individual gene names in the gene group are listed on the right-hand side of the map and functional annotation terms are presented on the lower side of the map. Annotation cluster 1 for immunoglobulin-like molecules, annotation cluster 2 for immunoglobulin heavy constant region, annotation cluster 3 for immunoglobulin variable region, annotation cluster 4 for structural constituent of cytoskeleton, and annotation cluster 5 for cell adhesion. Green rectangle, corresponding gene-annotation association positively reported in DAVID; black rectangle, corresponding gene-annotation association not yet reported in DAVID.

One hundred and forty genes with a signal log ratio (SLR) cutoff of at least 1 were overexpressed in tissues with fibrotic lesions versus emphysematous lesions (Additional file 1: Table S1). When enrichment analysis was applied to this set of genes, five functional annotation clusters emerged (Figure 5); the most highly enriched was a cluster of immunoglobulin-like molecules with an enrichment score of 13.13. The other clusters were based on annotations of immunoglobulin heavy constant region (score of 5.57), immunoglobulin variable region (score of 3.55), structural constituent of cytoskeleton (score of 3.55), and cell adhesion (score of 2.94).

### Gene functional classification for emphysematous lesions in CPFE

**Figure 6:**
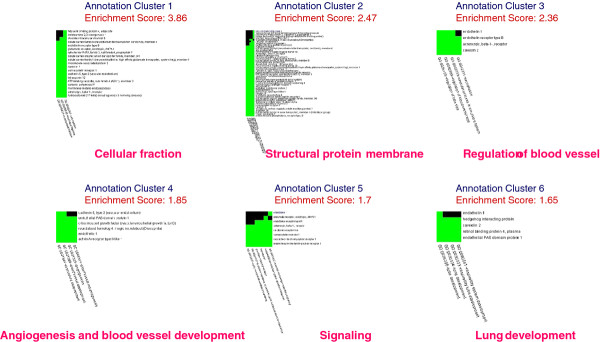
**Highly related genes and corresponding annotations in emphysematous lesions of combined pulmonary fibrosis and emphysema.** Two-dimensional view maps of the relationships between highly related genes and their corresponding annotations in emphysematous lesions of combined pulmonary fibrosis and emphysema (CPFE) using the Gene Functional Classification Tool ( http://david.abcc.ncifcrf.gov/gene2gene.jsp) from the Database for Annotation, Visualization and Integrated Discovery (DAVID; http://david.abcc.ncifcrf.gov/home.jsp). Annotation cluster 1 for cellular fraction, annotation cluster 2 for proteins in structure of membrane, annotation cluster 3 for regulation of blood vessel, annotation cluster 4 for angiogenesis and blood vessel development, annotation cluster 5 for signaling, and annotation cluster 6 for lung development. Green rectangle, corresponding gene-annotation association positively reported in DAVID; black rectangle, corresponding gene-annotation association not yet reported in DAVID.

After application of an analogous analysis process, 148 genes were identified as overexpressed in emphysematous tissues relative to fibrotic tissues (Additional file 2: Table S2). Of the six functional annotation clusters in the emphysematous lesion dataset (Figure 6), the cellular fraction cluster was the most enriched, with a score of 3.86. The other functional clusters were proteins in structure of membrane (score of 2.47), regulation of blood vessel (score of 2.36), angiogenesis and blood vessel development (score of 1.85), second-messenger-mediated signaling (score of 1.7), and lung development (score of 1.65).

## Discussion

The present study demonstrates in patients with CPFE in which pulmonary fibrosis and emphysema coexisted that the genes overexpressed in the fibrotic lesions were functionally different from those overexpressed in the emphysematous lesions. The genes overexpressed in the fibrotic lesions were annotated as contributing to functions involving immunoglobulin, the structural constituents of the cytoskeleton, and cellular adhesion – biological functions that are related to the pathophysiology of fibrosis. The set of genes overexpressed in the emphysematous lesions were annotated as associated with cell membrane structures, vascular growth and biology, second-messenger-mediated signaling, and lung development – all processes that contribute to the destruction and repair of cells, vessels, and the lung. These annotation-based clusters suggest that functionally different genes expressed in susceptible fields of the lungs eventually contribute to the formation of either fibrosis or emphysema, depending on the pattern of gene expression in that field.

The major finding of the present investigation is that genes belonging to the clusters enriched for immunoglobulin-like molecules, the immunoglobulin constant region, and the immunoglobulin variable region were markedly expressed in the fibrotic lesions. The roles of autoimmunity in interstitial lung diseases (fibrosis in pathology) associated with connective tissue disorders such as systemic sclerosis, systemic lupus erythematosus, and rheumatoid arthritis are well established [6]. Autoimmunity has been demonstrated to be one of the mechanisms of alveolar injury responsible for IPF [6,7]; the presence of autoantibodies in the sera of patients with IPF has been demonstrated elsewhere [8,9]. Additionally, the initial hypothesis for the mechanism of pulmonary fibrosis was that pulmonary inflammation was a prominent and necessary feature of the fibrotic process [10]. Those genes involved with immunoglobulin were probably expressed by the inflammatory cells in the infiltrates that were clearly presented in the fibrotic tissues. However, to date no candidate genes related to immunoglobulin-like molecules or members of the immunity system annotation cluster have been reported to be associated with lung fibrosis either in case–control association studies [11,12] or in studies based on human genome oligonucleotide microarrays [13]. Our observations provide the first evidence of a positive association of the immune system and pulmonary fibrosis at the genetic level. This major finding further supports the inflammation hypothesis of pulmonary fibrosis [10,14].

Different from other findings in which the results of gene expression in pulmonary fibrosis were resulted from the comparisons of the gene expression datasets between fibrous tissues and healthy lung tissues [13] or hypersensitivity pneumonitis lung tissues [15], we found that gene members of the structural constituent of cytoskeleton (keratin, claudin, dystonin) annotation cluster were overexpressed in CPFE fibrotic lesions versus emphysematous lesions. This observation provides genetic evidence in support of the latest mechanism of pulmonary fibrosis [16], which postulates that the losses of epithelial cells, endothelial cells, and alveolar–capillary barrier basement membrane integrity contribute to the pathogenesis of pulmonary fibrosis. Furthermore, we identified overexpression of the gene members of the cell adhesion annotation cluster in the fibrosis lesions of CPFE. Previously, cell adhesion molecules were shown to be expressed in the lungs of patients with IPF [17]. Cell–cell and cell–ECM interactions are critical for the pathogenesis of pulmonary fibrosis [18]. One of the essential mechanisms by which cells interact with the microenvironment is through the expression of cell adhesion molecules [18]. Inflammatory cells may adhere to and injure lung parenchymal cells through binding to intercellular adhesion molecule 1. After initial injury, the abnormalities in interepithelial adhesion interactions, the interactions of ECM molecules with both epithelial cells and fibroblasts, and the disordered healing process eventually result in fibrosis [18].

Regarding the genes expressed in emphysematous lesions in CPFE, our results showed that the genes involved in cellular and membrane fractions, genes encoding proteins in membranes, genes related to blood vessel size, development, and angiogenesis, and genes related to second-messenger-mediated signaling were overexpressed versus fibrotic lesions of CPFE. We propose that the overexpression of genes with functions contributing to the cellular fraction and membrane structure may lead to the injury of pulmonary alveoli to cause emphysema. In addition, our observation of the overexpressed genes regulating blood vessel size, development, and angiogenesis in the emphysematous lesions in CPFE may explain the genetic susceptibility to high prevalence (up to 47%) of pulmonary hypertension [1] in CPFE. We suggest that the overexpression of these vascular biology-related genes orchestrates the development and progression of pulmonary hypertension in CPFE. This hypertension may be a clinical characteristic that distinguishes CPFE from severe emphysema without fibrosis; the latter has a lower frequency of pulmonary hypertension than CPFE [1] and expresses downregulated endothelium-related genes [5].

The main limitation of the present study is the very small sample size, with only three patients with CPFE, which occurred because lung tissue specimens were only occasionally available following invasive operations for other diseases; for example, lung cancer. Further study with a larger sample size is necessary to confirm our observations. Nevertheless, we believe that the present results motivate a new direction for understanding the coexistence of fibrosis and emphysema in CPFE.

## Conclusions

In summary, we have demonstrated that gene expression differs between fibrotic and emphysematous lesions in CPFE. In the fibrotic lesions, genes associated with the immune system are highly expressed, while genes related to the cellular fraction, membrane biology, and vascular biology are highly expressed in emphysematous lesions. We propose that the development of coexistent fibrotic and emphysematous lesions in CPFE is implemented by these different patterns of gene expression.

## Methods

### Patients and specimens

According to the characteristics of CPFE addressed by Cottin and colleagues [1], six patients were selected for the current study. All six patients were originally diagnosed with lung cancer and high-resolution computed tomography showed not only the cancerous lesions but also emphysematous lesions in the upper zones and fibrotic lesions in the lower zones. The fibrotic and emphysematous lesions were located within the resected lobes. Further pulmonary function tests also indicated that impairment of carbon monoxide diffusing capacity and subnormal spirometry were present in these patients. CPFE was also diagnosed for these patients. Lung lobectomy was thought to be the optimal treatment for the lung cancer after consulting with chest surgeons. Specimens of fibrotic lesions and emphysematous lesions in lung tissues were sampled from the resected lobes of the six patients. Immediately after the lobectomy, the lung tissues with fibrosis and emphysema were separated from the resected lobes, and the lung tissue adjacent to each specimen was cut for histological confirmation of the absence of cancerous cells. The pathological types of fibrosis and emphysema were examined and confirmed after H & E staining by a pathologist who was blinded to the purpose of the study. This study was approved by the Ethics Committee of Shinshu University School of Medicine, and written informed consent was obtained from all patients.

### RNA isolation

Lung tissue specimens were immediately frozen in dry ice and stored at −80°C. RNA was extracted from the lung tissues using TRIzol Reagent (Life Technologies, Rockville, MD, USA) according to the manufacturer’s protocol. RNA for microarray analysis was purified using the RNeasy MinElute Cleanup Kit (QIAGEN, Valencia, CA, USA) according to the manufacturer’s instructions. RNA quality was assessed with an Agilent 2100 Bioanalyzer (Agilent Technologies, Palo Alto, CA, USA).

### GeneChip expression preprocessing

In accordance with the standard Affymetrix protocol from the GeneChip Expression Analysis Technical Manual Revision 5 [19], 2 μg total RNA was processed, biotinylated, fragmented, and hybridized to the GeneChip Human Genome U133 Plus 2.0 Array (Affymetrix, Santa Clara, CA, USA). The Human Genome U133 Plus 2.0 chips consist of 54,765 probe sets and provide comprehensive coverage of the transcribed human genome on a single array, allowing the analysis of more than 47,000 transcripts and variants, including 38,500 well-characterized human genes plus approximately 6,500 new genes [20]. Immediately following hybridization, the probe array underwent an automated washing and staining protocol on the Affymetrix Fluidics Station 450. The prepared GeneChips were then scanned using the Affymetrix GeneChip Scanner 3000. Each probe array was scanned twice and the software calculated an average of the two images, defined the probe cells, and computed the intensity for each cell. The double scan improved assay sensitivity and reduced background noise. Each complete probe array image was stored in a separate data file.

### Data processing

The scanned images were analysed with GeneChip Operating Software version 1.4 (Affymetrix 690036) and Microarray Suite version 5.0 (Affymetrix). The advantages of the Microarray Suite include the associated *P* values indicating statistical significance for detection and change calls, the confidence limits being associated with expression change values, and the negative expression values being eliminated [21]. The profiling dataset from the emphysematous lesions was used as background when extracting the differentially expressed genes in the fibrotic lesions, and the data from the fibrotic lesions were used as background when extracting the differentially expressed genes in the emphysematous lesions. The trimmed mean target intensity of each array was set to 500. Differentially expressed genes were extracted using DNA Microarray Viewer (Kurabo, Osaka, Japan).

All microarray data of the individual lung tissue of the six specimens are freely available through the Gene Expression Omnibus repository [GEO:GSE38934].

### Data analysis

The paired *t* test was performed for comparison of the detected signals between fibrotic and emphysematous lesions on the microarray data. The volcano plot was derived from the summary results of the paired *t* test to represent the point of intersection of fold-change (emphysema vs. fibrosis) relative to *P* value (emphysema vs. fibrosis). Clustering analysis was then carried out with log-transformed average signals of both fibrotic and emphysematous lesions on the microarray data and the heat map was built (Avadis 4.3; Strand Scientific Intelligence, San Francisco, CA, USA), according to the result of the hierarchical clustering analysis.

The SLR algorithm measures the magnitude and direction of the change between transcript levels of the experimental and background chips. A SLR of 1 represents a twofold increase in the abundance of an mRNA, and a value of −1 represents a twofold reduction in transcript expression. In the present analysis, we extracted genes with SLR >1 and SLR <1 for the fibrotic/emphysematous and emphysematous/fibrotic ratios, respectively. We then systematically annotated the large list of genes according to their biological functions using bioinformatics resources from the Database for Annotation, Visualization and Integrated Discovery [22], which provides the ability to explore and view functionally related genes together, as a unit, to concentrate on the large biological network rather than at the level of an individual gene [9]. Condensing large gene lists into biologically meaningful modules greatly improves the ability to assimilate large amounts of information and thus switches functional annotation analysis from a gene-centric analysis to a biological module-centric analysis [9]. Since an enrichment score of 1.3 is equivalent to 0.05 on the nonlog scale, we focused on gene clusters with scores ≥1.3 to address genes hypothetically involved in the phenotypes of the examined tissues [9].

## Abbreviations

CPFE: Combined pulmonary fibrosis and emphysema; ECM: Extracellular matrix; H & E: Hematoxylin and eosin; IPF: Idiopathic pulmonary fibrosis; SLR: Signal log ratio.

## Competing interests

The authors declare that they have no competing interests.

## Authors’ contributions

MH conceived of the study, participated in its design and coordination, and drafted the manuscript. MI collected samples, performed the experiments and statistical analysis, and analyzed the data. YD analyzed the data and drafted the manuscript. AU, YK, and MY were responsible for the clinical data of the patients for diagnosis. KK conceived and designed the study. All authors read and approved the final manuscript.

## Supplementary Material

Additional file 1 **Table S1.** One hundred and forty genes with signal log ratio over 1 were overexpressed in lung tissues with fibrotic lesions versus tissues with emphysematous lesions. Click here for file

Additional file 2 **Table S2. **One hundred and forty eight genes with signal log ratio less than 1 were overexpressed in lung tissues with emphysematous lesions versus tissues with fibrotic lesions. Click here for file
