# Supplementary material for: Comparison of gene expression profiling between lung fibrotic and emphysematous tissues sampled from patients with combined pulmonary fibrosis and emphysema
Source: Fibrogenesis Tissue Repair. 2012 Oct 1;5:17. doi: 10.1186/1755-1536-5-17 (PMC3541270; doi:10.1186/1755-1536-5-17)
Supplement: Additional file 1 — Table S1. One hundred and forty genes with signal log ratio over 1 were overexpressed in lung tissues with fibrotic lesions versus tissues with emphysematous lesions. [file 1755-1536-5-17-S1.docx]

Comparison of gene expression profiling between lung fibrotic and emphysematous tissues sampled from patients with combined pulmonary fibrosis and emphysema

**Masayuki Hanaoka*, Michiko Ito, Yunden Droma, Atsuhito Ushiki, Yoshiaki Kitaguchi, Masanori Yasuo, Keishi Kubo**

First Department of Medicine, Shinshu University School of Medicine, 3-1-1 Asahi, Matsumoto, Japan

*Corresponding author

E-mail addresses: [masayuki@shinshu-u.ac.jp](mailto:masayuki@shinshu-u.ac.jp)

Data deposition: The data reported in this paper have been deposited in the Gene Expression Omnibus database: <http://www.ncbi.nlm.nih.gov/geo/query/acc.cgi?acc=GSE38934>

Additional file 1:

Table S1. One hundred and forty genes with signal log ratio over 1 were overexpressed in lung tissues with fibrotic lesions versus tissues with emphysematous lesions

Table S1. One hundred and forty genes with signal log ratio over 1 were overexpressed in lung tissues with fibrotic lesions versus tissues with emphysematous lesions

| **Probe Name^*^** | **SLR^†^** | **P-value^‡^** | **Gene Title** | **Gene Symbol** | **Chromosomal Location** | **Gene Ontology Molecular Function** |
| --- | --- | --- | --- | --- | --- | --- |
| 201820_at | 2.7 | 2.0.E-05 | keratin 5 | KRT5 | chr12q12-q13 | structural constituent of cytoskeleton |
| 204136_at | 1.7 | 8.4.E-05 | collagen, type VII, alpha 1 | COL7A1 | chr3p21.1 | G-protein-coupled receptor binding |
| 204259_at | 2.5 | 4.9.E-05 | matrix metallopeptidase 7 | MMP7 | chr11q21-q22 | metalloendopeptidase activity |
| 204268_at | 3.3 | 2.0.E-05 | S100 calcium binding protein A2 | S100A2 | chr1q21 | calcium ion binding |
| 204269_at | 1.4 | 9.4.E-05 | pim-2 oncogene | PIM2 | chrXp11.23 | nucleotide binding |
| 204455_at | 1.9 | 7.5.E-05 | dystonin | DST | chr6p12.1 | actin binding |
| 204469_at | 1.9 | 4.7.E-04 | protein tyrosine phosphatase, | PTPRZ1 | chr7q31.3 | phosphoprotein phosphatase activity |
| 204475_at | 4.1 | 2.0.E-05 | matrix metallopeptidase 1 | MMP1 | chr11q22.3 | metalloendopeptidase activity |
| 204532_x_at | 2.3 | 6.6.E-04 | UDP glucuronosyltransferase 1 family, polypeptide A1 | UGT1A1 | chr2q37 | glucuronosyltransferase activity |
| 204734_at | 2.9 | 2.9.E-05 | keratin 15 | KRT15 | chr17q21.2 | structural constituent of cytoskeleton |
| 204855_at | 2.8 | 4.4.E-05 | serpin peptidase inhibitor, clade B (ovalbumin), member 5 | SERPINB5 | chr18q21.3 | serine-type endopeptidase inhibitor activity |
| 205157_s_at | 1.7 | 2.0.E-05 | keratin 17 | KRT17 | chr17q12-q21 | structural constituent of cytoskeleton |
| 205267_at | 2.2 | 2.6.E-05 | POU class 2 associating factor 1 | POU2AF1 | chr11q23.1 | DNA binding |
| 205513_at | 2.2 | 2.3.E-05 | transcobalamin I (vitamin B12 binding protein, R binder family) | TCN1 | chr11q11-q12 | cobalt ion transmembrane transporter activityinferred from electronic annotation |
| 205901_at | 1.4 | 6.7.E-04 | prepronociceptin | PNOC | chr8p21 | opioid peptide activity |
| 205903_s_at | 1.5 | 4.4.E-04 | potassium intermediate　calcium-activated channel, subfamily N, member 3 | KCNN3 | chr1q21.3 | ion channel activity |
| 205997_at | 1.8 | 3.1.E-05 | ADAM metallopeptidase domain 28 | ADAM28 | chr8p21.2 | metalloendopeptidase activity |
| 206032_at | 2.1 | 2.1.E-04 | desmocollin 3 | DSC3 | chr18q12.1 | calcium ion binding |
| 206033_s_at | 2.2 | 6.0.E-05 | desmocollin 3 | DSC3 | chr18q12.2 | calcium ion binding |
| 206094_x_at | 2.4 | 9.4.E-05 | UDP glucuronosyltransferase 1 family, polypeptide A1 | UGT1A1 | chr2q37 | UDP-glycosyltransferase activity |
| 206121_at | 1.7 | 7.6.E-05 | adenosine monophosphate deaminase 1 (isoform M) | AMPD1 | chr1p13 | AMP deaminase activity |
| 206150_at | 1.3 | 3.8.E-05 | CD27 molecule | CD27 | chr12p13 | receptor activity |
| 206165_s_at | 2.4 | 8.8.E-05 | chloride channel accessory 2 | CLCA2 | chr1p31-p22 | chloride channel activity |
| 206276_at | 3.8 | 6.5.E-05 | lymphocyte antigen 6 complex, locus D | LY6D | chr8q24-qter | protein binding |
| 206478_at | 2.3 | 4.3.E-05 | KIAA0125 | KIAA0125 | chr14q32.33 | --- |
| 206513_at | 1.2 | 3.7.E-04 | absent in melanoma 2 | AIM2 | chr1q22 | protein binding |
| 206641_at | 2.0 | 3.6.E-05 | tumor necrosis factor receptor superfamily, member 17 | TNFRSF17 | chr16p13.1 | receptor activity |
| 206686_at | 1.4 | 1.2.E-03 | pyruvate dehydrogenase kinase, isozyme 1 | PDK1 | chr2q31.1 | two-component sensor activity |
| 207126_x_at | 2.9 | 2.3.E-04 | UDP glucuronosyltransferase 1 family, polypeptide A1 | UGT1A1 | chr2q37 | glucuronosyltransferase activity |
| 207734_at | 1.3 | 8.2.E-05 | lymphocyte transmembrane adaptor 1 | LAX1 | chr1q32.1 | protein binding |
| 208153_s_at | 1.8 | 2.4.E-04 | FAT tumor suppressor homolog 2 (Drosophila) | FAT2 | chr5q32-q33 | calcium ion binding |
| 208269_s_at | 1.4 | 7.6.E-04 | ADAM metallopeptidase domain 28 | ADAM28 | chr8p21.2 | metalloendopeptidase activity |
| 208596_s_at | 2.7 | 1.4.E-04 | UDP glucuronosyltransferase 1 family, polypeptide A1 | UGT1A1 | chr2q37 | UDP-glycosyltransferase activity |
| 209125_at | 2.6 | 3.9.E-05 | keratin 6A | KRT6A | chr12q12-q13 | structural molecule activity |
| 209374_s_at | 1.3 | 3.9.E-05 | immunoglobulin heavy constant mu | IGHM | chr14q32.33 | antigen binding |
| 209863_s_at | 1.6 | 5.6.E-05 | tumor protein p63 | TP63 | chr3q28 | DNA binding |
| 209875_s_at | 2.3 | 2.0.E-05 | secreted phosphoprotein 1 | SPP1 | chr4q21-q25 | cytokine activity |
| 210387_at | 1.7 | 1.2.E-04 | histone cluster 1, H2bg | HIST1H2BG | chr6p21.3 | DNA binding |
| 211633_x_at | 1.8 | 5.6.E-05 | Immunoglobulin heavy constant gamma 1 (G1m marker) | IGHG1 | chr14q32.33 | antigen binding |
| 211634_x_at | 2.2 | 3.0.E-05 | immunoglobulin heavy constant | IGHM | chr14q32.33 | antigen binding |
| 211635_x_at | 2.3 | 1.7.E-04 | immunoglobulin heavy locus | IGHA1 | chr14q32.33 | antigen binding |
| 211637_x_at | 2.0 | 2.6.E-04 | immunoglobulin heavy locus | GHA0 | chr14q32.33 | antigen binding |
| 211639_x_at | 1.7 | 1.4.E-04 | immunoglobulin heavy locus | GHA1 | chr14q32.33 | antigen binding |
| 211640_x_at | 2.4 | 1.5.E-04 | immunoglobulin heavy constant gamma 1 (G1m marker) | IGHG1 | chr14q32.33 | antigen binding |
| 211641_x_at | 1.6 | 9.2.E-05 | immunoglobulin heavy locus | IGHA1 | chr14q32.33 | antigen binding |
| 211643_x_at | 1.9 | 2.0.E-05 | immunoglobulin kappa locus | IGHA2 | chr2p11.2 | antigen binding |
| 211644_x_at | 1.9 | 2.0.E-05 | immunoglobulin kappa locus | IGHA2 | chr2p11.2 | antigen binding |
| 211645_x_at | 1.8 | 2.0.E-05 | --- | --- | --- | --- |
| 211650_x_at | 1.8 | 4.9.E-05 | immunoglobulin heavy constant alpha 1 | IGHA1 | chr14q32.33 | antigen binding |
| 211798_x_at | 1.8 | 5.5.E-05 | immunoglobulin lambda joining 3 | IGLJ3 | chr22q11.1-q11.2 | --- |
| 211868_x_at | 1.5 | 5.2.E-04 | immunoglobulin heavy locus | IGHA1 | chr14q32.33 | antigen binding |
| 211881_x_at | 2.0 | 5.2.E-05 | immunoglobulin lambda joining 3 | IGLJ3 | chr22q11.1-q11.2 | --- |
| 211908_x_at | 1.9 | 4.3.E-04 | Immunoglobulin heavy constant gamma 1 (G1m marker) | IGHG1 | chr14q32.33 | antigen binding |
| 212236_x_at | 1.4 | 2.4.E-05 | keratin 17 | KRT17 | chr17q12-q21 | structural molecule activity |
| 214580_x_at | 3.5 | 6.0.E-04 | keratin 6A | KRT6A | chr12q12-q13 | structural molecule activity |
| 214768_x_at | 1.9 | 2.1.E-05 | Family with sequence similarity 20, member B | FAM20B | chr1q25 | --- |
| 214777_at | 1.4 | 2.0.E-05 | immunoglobulin kappa variable 4-1 | IGKV4-1 | chr2p12 | antigen binding |
| 214916_x_at | 1.6 | 2.4.E-05 | immunoglobulin heavy locus | IGHA1 | chr14q32.33 | antigen binding |
| 214973_x_at | 1.8 | 2.0.E-05 | immunoglobulin heavy constant delta | IGHD | chr14q32.33 | antigen binding |
| 215035_at | 2.7 | 5.9.E-04 | immunoglobulin lambda variable 6-57 | IGLV6-57 | chr22q11.2 | --- |
| 215118_s_at | 1.5 | 5.7.E-04 | Immunoglobulin heavy constant gamma 1 (G1m marker) | IGHG1 | chr14q32.33 | antigen binding |
| 215125_s_at | 3.5 | 3.7.E-04 | UDP glucuronosyltransferase 1 family, polypeptide A1 | UGT1A1 | chr2q37 | UDP-glycosyltransferase activity |
| 215176_x_at | 1.4 | 2.0.E-05 | similar to hCG26659 | LOC100291464 | --- | antigen binding |
| 215214_at | 2.2 | 2.0.E-05 | Immunoglobulin lambda locus | IGL@ | chr22q11.1-q11.2 | --- |
| 215565_at | 2.0 | 1.9.E-04 | hypothetical protein LOC100289053 | LOC100289053 | chr2p23.3 | calcium ion binding |
| 215777_at | 1.4 | 8.9.E-05 | immunoglobulin lambda variable 4-60 | IGLV4-60 | chr22q11.2 | --- |
| 215949_x_at | 1.5 | 6.1.E-05 | immunoglobulin heavy constant | IGHM 4 | chr14q32.33 | antigen binding |
| 216207_x_at | 1.5 | 3.0.E-05 | immunoglobulin kappa constant | IGKC | chr2p12 | antigen binding |
| 216365_x_at | 2.3 | 2.0.E-05 | immunoglobulin lambda locus | IGL@ | chr22q11.1-q11.2 | --- |
| 216401_x_at | 1.8 | 3.1.E-05 | similar to Ig kappa chain V-I region HK102 precursor | LOC652493 | --- | antigen binding |
| 216412_x_at | 2.5 | 2.5.E-05 | immunoglobulin lambda locus | IGL@ | chr22q11.1-q11.2 | --- |
| 216430_x_at | 2.2 | 1.9.E-04 | immunoglobulin lambda locus | IGL@ | chr22q11.1-q11.2 | --- |
| 216491_x_at | 2.1 | 2.5.E-04 | immunoglobulin heavy constant | IGHM | chr14q32.33 | antigen binding |
| 216510_x_at | 1.9 | 2.0.E-04 | immunoglobulin heavy constant alpha 1 | IGHA1 | chr14q32.33 | antigen binding |
| 216541_x_at | 2.3 | 3.9.E-04 | immunoglobulin heavy constant gamma 1 (G1m marker) | IGHG1 | chr14q32.33 | antigen binding |
| 216542_x_at | 1.8 | 2.1.E-04 | immunoglobulin heavy constant alpha 1 | IGHA1 | chr14q32.33 | antigen binding |
| 216557_x_at | 1.8 | 6.0.E-05 | immunoglobulin heavy constant alpha 1 | IGHA1 | chr14q32.33 | antigen binding |
| 216560_x_at | 2.3 | 2.7.E-05 | immunoglobulin lambda locus | IGL@ | chr22q11.1-q11.2 | --- |
| 216576_x_at | 1.8 | 4.1.E-05 | immunoglobulin kappa locus | IGK@ | chr2p12 | antigen binding |
| 216853_x_at | 2.1 | 3.1.E-05 | immunoglobulin lambda variable 3-19 | IGLV3-19 | chr22q11.2 | --- |
| 216984_x_at | 1.7 | 7.6.E-05 | immunoglobulin lambda locus | IGL@ | chr22q11.1-q11.2 | --- |
| 217084_at | 2.1 | 6.5.E-04 | immunoglobulin heavy constant alpha 1 | IGHA1 | chr14q32.33 | antigen binding |
| 217148_x_at | 1.9 | 2.0.E-05 | similar to Ig lambda chain | LOC100293440 | --- | --- |
| 217157_x_at | 1.6 | 3.5.E-05 | immunoglobulin kappa locus | IGK@ | chr2p12 | antigen binding |
| 217179_x_at | 2.0 | 2.8.E-05 | --- | --- | --- | antigen binding |
| 217198_x_at | 1.7 | 3.4.E-04 | immunoglobulin heavy locus | IGH@ | chr14q32.33 | antigen binding |
| 217227_x_at | 1.9 | 2.0.E-05 | immunoglobulin lambda locus | IGL@ | chr22q11.1-q11.2 / | --- |
| 217235_x_at | 1.9 | 2.5.E-05 | immunoglobulin lambda locus | IGL@ | chr22q11.1-q11.2 | --- |
| 217258_x_at | 2.4 | 2.0.E-05 | immunoglobulin lambda locus | IGL@ | chr22q11.1-q11.2 | --- |
| 217281_x_at | 1.9 | 2.3.E-05 | immunoglobulin heavy locus | IGH@ | chr14q32.33 | antigen binding |
| 217378_x_at | 1.7 | 6.0.E-05 | similar to hCG26659 | LOC100130100 | chr2q13 | --- |
| 217384_x_at | 2.3 | 4.4.E-04 | immunoglobulin heavy variable 3-23 | IGHV3-23 | chr14q32.33 | antigen binding |
| 217480_x_at | 1.7 | 3.5.E-05 | similar to Ig kappa chain | LOC100287723 | chr10q11.21 | --- |
| 217528_at | 3.6 | 1.5.E-04 | chloride channel accessory 2 | CLCA2 | chr1p31-p22 | chloride channel activity |
| 218002_s_at | 2.2 | 3.6.E-05 | chemokine (C-X-C motif) ligand 14 | CXCL14 | chr5q31 | cytokine activity |
| 218468_s_at | 1.7 | 2.0.E-05 | gremlin 1, cysteine knot superfamily, homolog | GREM1 | chr15q13-q15 | cytokine activity |
| 218469_at | 1.6 | 2.0.E-05 | gremlin 1, cysteine knot superfamily, homolog | GREM1 | chr15q13-q15 | cytokine activity |
| 218960_at | 2.8 | 2.9.E-04 | transmembrane protease, serine 4 | TMPRSS4 | chr11q23.3 | catalytic activity |
| 219118_at | 1.4 | 2.0.E-05 | FK506 binding protein 11, 19 kDa | FKBP11 | chr12q13.12 | peptidyl-prolyl cis-trans isomerase activity |
| 219159_s_at | 1.7 | 4.0.E-04 | SLAM family member 7 | SLAMF7 | chr1q23.1-q24.1 | receptor activity |
| 219936_s_at | 2.5 | 1.4.E-04 | G protein-coupled receptor 87 | GPR87 | chr3q24 | signal transducer activity |
| 220306_at | 1.5 | 6.2.E-05 | family with sequence similarity 46, member C | FAM46C | chr1p12 | --- |
| 220338_at | 1.3 | 3.4.E-04 | Ral GEF with PH domain and SH3 binding motif 2 | RALGPS2 | chr1q25.2 | guanyl-nucleotide exchange factor activity |
| 221286_s_at | 2.1 | 3.4.E-05 | hypothetical protein MGC29506 | MGC29506 | chr5q23-q31 | --- |
| 222068_s_at | 2.6 | 3.6.E-05 | leucine rich repeat containing 50 | LRRC50 | chr16q23.3-q24.1 | protein binding |
| 222484_s_at | 2.3 | 2.0.E-05 | chemokine (C-X-C motif) ligand 14 | CXCL14 | chr5q31 | cytokine activity |
| 222549_at | 1.2 | 2.2.E-05 | claudin 1 | CLDN1 | chr3q28-q29 | structural molecule activity |
| 222838_at | 1.6 | 2.0.E-05 | SLAM family member 7 | SLAMF7 | chr1q23.1-q24.1 | receptor activity |
| 223565_at | 2.1 | 2.1.E-05 | hypothetical protein MGC29506 | MGC29506 | chr5q23-q31 | --- |
| 224342_x_at | 1.9 | 2.0.E-05 | immunoglobulin lambda locus | IGL@ | chr22q11.1-q11.2 | antigen binding |
| 224404_s_at | 2.0 | 2.0.E-05 | Fc receptor-like 5 | FCRL5 | chr1q21 | receptor activity |
| 224406_s_at | 1.5 | 7.1.E-04 | Fc receptor-like 5 | FCRL5 | chr1q21 | receptor activity |
| 226150_at | 1.1 | 2.8.E-05 | phosphatidic acid phosphatase type 2 domain containing 1B | PPAPDC1B | chr8p11.23 | catalytic activity |
| 226452_at | 1.5 | 2.4.E-05 | pyruvate dehydrogenase kinase, isozyme 1 | PDK1 | chr2q31.1 | two-component sensor activity |
| 227224_at | 1.4 | 2.7.E-05 | Ral GEF with PH domain and SH3 binding motif 2 | RALGPS2 | chr1q25.2 | guanyl-nucleotide exchange factor activity |
| 229147_at | 1.7 | 1.3.E-04 | --- | --- | --- | --- |
| 229656_s_at | 1.5 | 1.9.E-04 | echinoderm microtubule associated protein like 6 | EML6 | chr2p16.2-p16.1 | --- |
| 229721_x_at | 1.8 | 2.0.E-05 | Der1-like domain family, member 3 | DERL3 | chr22q11.23 | protein binding |
| 230030_at | 1.7 | 6.9.E-05 | heparan sulfate 6-O-sulfotransferase 2 | HS6ST2 | chrXq26.2 | sulfotransferase activity |
| 230128_at | 3.2 | 2.3.E-04 | Immunoglobulin lambda locus | IGL@ | chr22q11.1-q11.2 | --- |
| 231145_at | 2.2 | 3.5.E-04 | --- | --- | --- | --- |
| 231647_s_at | 2.4 | 2.0.E-05 | Fc receptor-like 5 | FCRL5 | chr1q21 | receptor activity |
| 233413_at | 3.4 | 4.8.E-05 | --- | --- | --- | --- |
| 233463_at | 2.4 | 1.2.E-04 | Ras association (RalGDS/AF-6) domain family member 6 | RASSF6 | chr4q13.3 | protein binding |
| 234366_x_at | 1.7 | 2.2.E-05 | immunoglobulin lambda locus | IGL@ | chr22q11.1-q11.2 | --- |
| 234390_x_at | 3.1 | 2.1.E-04 | Immunoglobulin heavy constant gamma 1 (G1m marker) | IGHG1 | chr14q32.33 | antigen binding |
| 234419_x_at | 2.3 | 1.7.E-04 | immunoglobulin heavy locus | IGH@ | chr14q32.33 | antigen binding |
| 234477_at | 3.1 | 4.5.E-04 | immunoglobulin heavy constant alpha 1 | IGHA1 | chr14q32.33 | antigen binding |
| 234764_x_at | 2.0 | 2.1.E-05 | Immunoglobulin lambda variable 1-44 | IGLV1-44 | chr22q11.2 | --- |
| 234792_x_at | 2.8 | 2.9.E-04 | immunoglobulin heavy constant alpha 1 | IGHA1 | chr14q32.33 | antigen binding |
| 234884_x_at | 1.9 | 1.4.E-03 | Immunoglobulin lambda locus | IGL@ | chr22q11.1-q11.2 | --- |
| 235075_at | 3.6 | 2.9.E-05 | desmoglein 3 (pemphigus vulgaris antigen) | DSG3 | chr18q12.1-q12.2 | calcium ion binding |
| 242680_at | 1.8 | 2.9.E-04 | --- | --- | --- | --- |
| 1552797_s_at | 2.2 | 3.3.E-05 | prominin 2 | PROM2 | chr2q11.1 | --- |
| 1556183_at | 1.7 | 8.0.E-05 | hypothetical LOC645784 | FLJ40330 | chr2p11.2 | --- |
| 1557383_a_at | 1.6 | 4.0.E-05 | --- | --- | --- | --- |
| 1558290_a_at | 1.2 | 2.1.E-04 | Pvt1 oncogene (non-protein coding) | PVT1 | chr8q24 | --- |
| 1561937_x_at | 1.4 | 3.1.E-05 | immunoglobulin heavy constant alpha 1 | IGHA1 | chr14q32.33 | antigen binding |
| 1568868_at | 1.8 | 9.9.E-04 | cytochrome P450, family 27, subfamily C, polypeptide 1 | CYP27C1 | chr2q14.3 | monooxygenase activity |
| 1569040_s_at | 2.0 | 2.0.E-05 | hypothetical LOC645784 | FLJ40330 | chr2p11.2 | --- |

^*^ Probe name in Affymetrix.

^†^ SLR = signal log ratio. The SLR algorithm measures the magnitude and direction of the change between signal levels of the fibrotic lesions versus emphysematous lesions.

^‡^ The significance of the signal changes between the fibrotic lesions versus emphysematous lesions. For example, P=2.0.E-05 indicates P= 2.0 x 10^-5^.

--- Unknown or undetected.
