# Supplementary material for: Comparison of gene expression profiling between lung fibrotic and emphysematous tissues sampled from patients with combined pulmonary fibrosis and emphysema
Source: Fibrogenesis Tissue Repair. 2012 Oct 1;5:17. doi: 10.1186/1755-1536-5-17 (PMC3541270; doi:10.1186/1755-1536-5-17)
Supplement: Additional file 2 — Table S2. One hundred and forty eight genes with signal log ratio less than 1 were overexpressed in lung tissues with emphysematous lesions versus tissues with fibrotic lesions. [file 1755-1536-5-17-S2.docx]

Table S2. One hundred and forty eight genes with signal log ratio less than 1 were overexpressed in lung tissues with emphysematous lesions versus tissues with fibrotic lesions

| **Probe Name^*^** | **SLR^†^** | **P-value^‡^** | **Gene Title** | **Gene Symbol** | **Chromosomal Location** | **Gene Ontology Molecular Function** |
| --- | --- | --- | --- | --- | --- | --- |
| 200879_s_at | -1.4 | 7.3.E-04 | endothelial PAS domain protein 1 | EPAS1 | chr2p21-p16 | DNA binding |
| 202524_s_at | -1.7 | 1.1.E-03 | sparc/osteonectin, cwcv and kazal-like domains proteoglycan 2 | SPOCK2 | chr10pter-q25.3 | calcium ion binding |
| 202674_s_at | -1.3 | 2.4.E-04 | LIM domain 7 | LMO7 | chr13q22.2 | actin binding |
| 203323_at | -1.2 | 2.4.E-04 | caveolin 2 | CAV2 | chr7q31.1 | protein binding |
| 203434_s_at | -3.3 | 2.4.E-04 | membrane metallo-endopeptidase | MME | chr3q25.1-q25.2 | metalloendopeptidase activity |
| 203435_s_at | -2.5 | 8.1.E-03 | membrane metallo-endopeptidase | MME | chr3q25.1-q25.2 | metalloendopeptidase activity |
| 203913_s_at | -1.9 | 1.5.E-03 | hydroxyprostaglandin dehydrogenase 15-(NAD) | HPGD | chr4q34-q35 | catalytic activity |
| 203914_x_at | -2.0 | 2.4.E-04 | hydroxyprostaglandin dehydrogenase 15-(NAD) | HPGD | chr4q34-q35 | catalytic activity catalytic activity |
| 203980_at | -2.4 | 2.4.E-04 | fatty acid binding protein 4, adipocyte | FABP4 | chr8q21 | transporter activity |
| 204073_s_at | -1.8 | 1.4.E-03 | chromosome 11 open reading frame 9 | C11orf9 | chr11q12-q13.1 | transcription factor activity |
| 204271_s_at | -1.4 | 2.4.E-04 | endothelin receptor type B | EDNRB | chr13q22 | endothelin-B receptor activity |
| 204273_at | -1.4 | 4.1.E-04 | endothelin receptor type B | EDNRB | chr13q22 | endothelin-B receptor activity |
| 204343_at | -1.2 | 2.4.E-04 | ATP-binding cassette, sub-family A (ABC1), member 3 | ABCA3 | chr16p13.3 | nucleotide binding |
| 204368_at | -1.1 | 5.7.E-04 | solute carrier organic anion transporter family, member 2A1 | SLCO2A1 | chr3q21 | transporter activity |
| 204519_s_at | -1.7 | 4.1.E-04 | plasma membrane proteolipid | PLLP | chr16q13 | ion channel activity |
| 204677_at | -1.2 | 4.1.E-04 | cadherin 5, type 2 (vascular endothelium) | CDH5 | chr16q22.1 | receptor binding |
| 204747_at | -1.2 | 7.3.E-04 | interferon-induced protein with tetratricopeptide repeats 3 | IFIT3 | chr10q24 | binding |
| 204787_at | -1.2 | 2.4.E-04 | V-set and immunoglobulin domain containing 4 | VSIG4 | chrXq12-q13.3 | protein binding |
| 204975_at | -1.4 | 2.4.E-04 | epithelial membrane protein 2 | EMP2 | chr16p13.2 | --- |
| 205019_s_at | -1.8 | 2.0.E-03 | vasoactive intestinal peptide receptor 1 | VIPR1 | chr3p22 | signal transducer activity |
| 205200_at | -1.8 | 4.1.E-04 | C-type lectin domain family 3, member B | CLEC3B | chr3p22-p21.3 | 3'-5'-exoribonuclease activity |
| 205462_s_at | -1.2 | 2.4.E-04 | hippocalcin-like 1 | HPCAL1 | chr2p25.1 | calcium ion binding |
| 205700_at | -1.5 | 1.6.E-03 | hydroxysteroid (17-beta) dehydrogenase 6 homolog (mouse) | HSD17B6 | chr12q13 | catalytic activity |
| 205751_at | -3.0 | 4.1.E-03 | SH3-domain GRB2-like 2 | SH3GL2 | chr9p22 | protein binding |
| 205765_at | -1.7 | 9.8.E-04 | cytochrome P450, family 3, subfamily A, polypeptide 5 | CYP3A5 | chr7q21.1 | monooxygenase activity |
| 205779_at | -1.1 | 2.0.E-02 | receptor (G protein-coupled) activity modifying protein 2 | RAMP2 | chr17q12-q21.1 | receptor activity |
| 205846_at | -1.6 | 2.4.E-04 | protein tyrosine phosphatase, receptor type, B | PTPRB | chr12q15-q21 | phosphoprotein phosphatase activity |
| 205856_at | -2.5 | 2.4.E-03 | solute carrier family 14 (urea transporter), member 1 | SLC14A1 | chr18q11-q12 | water transporter activity |
| 205866_at | -2.6 | 1.9.E-03 | ficolin (collagen/fibrinogen domain containing) 3 | FCN3 | chr1p36.11 | receptor binding |
| 206068_s_at | -1.3 | 2.4.E-04 | acyl-Coenzyme A dehydrogenase, long chain | ACADL | chr2q34-q35 | acyl-CoA dehydrogenase activity |
| 206208_at | -2.5 | 5.7.E-04 | carbonic anhydrase IV | CA4 | chr17q23 | carbonate dehydratase activity |
| 206209_s_at | -2.6 | 4.1.E-04 | carbonic anhydrase IV | CA4 | chr17q23 | carbonate dehydratase activity |
| 206311_s_at | -2.3 | 2.4.E-04 | phospholipase A2, group IB | PLA2G1B | chr12q23-q24.1 | phospholipase A2 activity |
| 206331_at | -1.5 | 2.4.E-04 | calcitonin receptor-like | CALCRL | chr2q32.1 | calcitonin gene-related polypeptide receptor activity |
| 206651_s_at | -2.8 | 2.4.E-04 | carboxypeptidase B2 | CPB2 | chr13q14.11 | carboxypeptidase activity |
| 206658_at | -1.9 | 5.7.E-04 | uroplakin 3B | UPK3B | chr7q11.2 | --- |
| 206701_x_at | -1.5 | 1.7.E-03 | endothelin receptor type B | EDNRB | chr13q22 | endothelin-B receptor activity |
| 206742_at | -1.9 | 4.5.E-03 | c-fos induced growth factor (vascular endothelial growth factor D) | FIGF | chrXp22.31 | receptor binding |
| 207069_s_at | -1.6 | 1.3.E-03 | SMAD family member 6 | SMAD6 | chr15q21-q22 | transcription factor activity |
| 207519_at | -3.8 | 1.9.E-03 | solute carrier family 6 (neurotransmitter transporter, serotonin), member 4 | SLC6A4 | chr17q11.1-q12 | neurotransmitter:sodium symporter activity |
| 209267_s_at | -1.1 | 2.4.E-04 | solute carrier family 39 (zinc transporter), member 8 | SLC39A8 | chr4q22-q24 | zinc ion binding |
| 209301_at | -1.6 | 2.4.E-04 | carbonic anhydrase II | CA2 | chr8q22 | carbonate dehydratase activity |
| 209369_at | -1.6 | 2.4.E-04 | annexin A3 | ANXA3 | chr4q13-q22 | phospholipase inhibitor activity |
| 209469_at | -2.0 | 2.4.E-04 | glycoprotein M6A | GPM6A | chr4q34 | calcium channel activity |
| 209470_s_at | -2.3 | 2.4.E-04 | glycoprotein M6A | GPM6A | chr4q34 | calcium channel activity |
| 209793_at | -1.9 | 5.7.E-04 | glutamate receptor, ionotropic, AMPA 1 | GRIA1 | chr5q33\|5q31.1 | receptor activity |
| 209840_s_at | -1.4 | 2.4.E-04 | leucine rich repeat neuronal 3 | LRRN3 | chr7q31.1 | protein binding |
| 209841_s_at | -1.4 | 2.4.E-04 | leucine rich repeat neuronal 3 | LRRN3 | chr7q31.1 | protein binding |
| 209904_at | -2.0 | 2.4.E-03 | troponin C type 1 | TNNC1 | chr3p21.3-p14.3 | calcium ion binding |
| 210029_at | -1.8 | 2.4.E-04 | indoleamine 2,3-dioxygenase 1 | IDO1 | chr8p12-p11 | tryptophan 2,3-dioxygenase activity |
| 210081_at | -2.6 | 2.4.E-04 | advanced glycosylation end product-specific receptor | AGER | chr6p21.3 | receptor activity |
| 210549_s_at | -1.3 | 1.3.E-03 | chemokine (C-C motif) ligand 23 | CCL23 | chr17q12 | cytokine activity |
| 210619_s_at | -1.5 | 2.4.E-04 | hyaluronoglucosaminidase 1 | HYAL1 | chr3p21.3-p21.2 | catalytic activity |
| 210815_s_at | -1.6 | 2.4.E-04 | calcitonin receptor-like | CALCRL | chr2q32.1 | calcitonin gene-related polypeptide receptor activity |
| 210838_s_at | -1.7 | 1.1.E-01 | activin A receptor type II-like 1 | ACVRL1 | chr12q11-q14 | nucleotide binding |
| 211548_s_at | -2.1 | 2.4.E-04 | hydroxyprostaglandin dehydrogenase 15-(NAD) | HPGD | chr4q34-q35 | catalytic activity |
| 211549_s_at | -1.9 | 2.4.E-04 | hydroxyprostaglandin dehydrogenase 15-(NAD) | HPGD | chr4q34-q35 | catalytic activity |
| 211887_x_at | -1.6 | 7.3.E-04 | macrophage scavenger receptor 1 | MSR1 | chr8p22 | receptor activity |
| 213317_at | -1.4 | 2.4.E-04 | chloride intracellular channel 5 | CLIC5 | chr6p21.1-p12.1 | ion channel activity |
| 213456_at | -3.1 | 2.4.E-04 | sclerostin domain containing 1 | SOSTDC1 | chr7p21.1 | protein binding |
| 213664_at | -1.4 | 2.4.E-04 | solute carrier family 1, member 1 | SLC1A1 | chr9p24 | L-glutamate transmembrane transporter activity statement |
| 214135_at | -2.3 | 2.4.E-04 | claudin 18 | CLDN18 | chr3q22.3 | structural molecule activity |
| 214234_s_at | -1.6 | 7.3.E-04 | cytochrome P450, family 3, subfamily A, polypeptide 5 | CYP3A5 | chr7q21.1 | monooxygenase activity |
| 214641_at | -1.5 | 2.0.E-03 | collagen, type IV, alpha 3 | COL4A3 | chr2q36-q37 | integrin binding |
| 215918_s_at | -1.4 | 2.4.E-04 | spectrin, beta, non-erythrocytic 1 | SPTBN1 | chr2p21 | actin binding |
| 216504_s_at | -1.3 | 2.4.E-04 | solute carrier family 39 (zinc transporter), member 8 | SLC39A8 | chr4q22-q24 | zinc ion binding |
| 217046_s_at | -2.6 | 5.7.E-04 | advanced glycosylation end product-specific receptor | AGER | chr6p21.3 | receptor activity |
| 217177_s_at | -1.3 | 2.4.E-04 | protein tyrosine phosphatase, receptor type, B | PTPRB | chr12q15-q21 | phosphoprotein phosphatase activity |
| 218711_s_at | -1.2 | 2.4.E-04 | serum deprivation response (phosphatidylserine binding protein) | SDPR | chr2q32-q33 | phosphatidylserine binding |
| 218995_s_at | -1.4 | 1.1.E-03 | endothelin 1 | EDN1 | chr6p24.1 | receptor binding |
| 219054_at | -1.8 | 2.4.E-04 | chromosome 5 open reading frame 23 | C5orf23 | chr5p13.3 | --- |
| 219140_s_at | -1.7 | 8.8.E-03 | retinol binding protein 4, plasma | RBP4 | chr10q23-q24 | transporter activity |
| 219230_at | -2.8 | 2.4.E-04 | transmembrane protein 100 | TMEM100 | chr17q22 | --- |
| 219274_at | -1.3 | 2.4.E-04 | tetraspanin 12 | TSPAN12 | chr7q31.31 | --- |
| 219295_s_at | -2.5 | 2.4.E-04 | procollagen C-endopeptidase enhancer 2 | PCOLCE2 | chr3q21-q24 | protein binding |
| 219529_at | -1.7 | 1.5.E-03 | chloride intracellular channel 3 | CLIC3 | chr9q34.3 | ion channel activity |
| 219584_at | -1.9 | 4.1.E-04 | phospholipase A1 member A | PLA1A | chr3q13.13-q13.2 | catalytic activity |
| 219612_s_at | -2.8 | 4.1.E-04 | fibrinogen gamma chain | FGG | chr4q28 | receptor binding |
| 219656_at | -1.1 | 2.3.E-03 | protocadherin 12 | PCDH12 | chr5q31 | calcium ion binding |
| 219689_at | -1.3 | 2.4.E-03 | sema domain, immunoglobulin domain (Ig), short basic domain, secreted, (semaphorin) 3G | SEMA3G | chr3p21.1 | receptor activity |
| 219694_at | -1.2 | 2.4.E-04 | family with sequence similarity 105, member A | FAM105A | chr5p15.2 | --- |
| 219866_at | -1.5 | 2.4.E-04 | chloride intracellular channel 5 | CLIC5 | chr6p21.1-p12.1 | ion channel activity |
| 219869_s_at | -1.3 | 2.4.E-04 | solute carrier family 39 (zinc transporter), member 8 | SLC39A8 | chr4q22-q24 | zinc ion binding |
| 220327_at | -1.1 | 2.4.E-04 | vestigial like 3 (Drosophila) | VGLL3 | chr3p12.1 | transcription regulator activity |
| 220994_s_at | -1.9 | 8.8.E-03 | syntaxin binding protein 6 (amisyn) | STXBP6 | chr14q12 | --- |
| 221132_at | -4.0 | 4.1.E-04 | claudin 18 | CLDN18 | chr3q22.3 | structural molecule activity |
| 221133_s_at | -2.4 | 4.1.E-04 | claudin 18 | CLDN18 | chr3q22.4 | structural molecule activity |
| 222121_at | -1.5 | 2.4.E-04 | Src homology 3 domain-containing guanine nucleotide exchange factor | SGEF | chr3q25.2 | guanyl-nucleotide exchange factor activity |
| 43511_s_at | -1.2 | 2.3.E-04 | --- | --- | --- | --- |
| 49111_at | -1.1 | 2.3.E-04 | --- | --- | --- | --- |
| 222717_at | -1.1 | 5.7.E-04 | serum deprivation response (phosphatidylserine binding protein) | SDPR | chr2q32-q33 | phosphatidylserine binding |
| 222802_at | -1.5 | 2.4.E-04 | endothelin 1 | EDN1 | chr6p24.1 | receptor binding |
| 223836_at | -2.3 | 1.9.E-02 | fibroblast growth factor binding protein 2 | FGFBP2 | chr4p16 | growth factor binding |
| 225078_at | -1.5 | 2.4.E-04 | epithelial membrane protein 2 | EMP2 | chr16p13.2 | --- |
| 225079_at | -1.5 | 2.4.E-04 | epithelial membrane protein 2 | EMP2 | chr16p13.2 | --- |
| 225540_at | -1.5 | 2.4.E-04 | microtubule-associated protein 2 | MAP2 | chr2q34-q35 | structural molecule activity |
| 225575_at | -1.1 | 4.1.E-04 | leukemia inhibitory factor receptor alpha | LIFR | chr5p13-p12 | receptor activity |
| 226028_at | -1.1 | 2.4.E-04 | roundabout homolog 4, magic roundabout (Drosophila) | ROBO4 | chr11q24.2 | receptor activity |
| 226145_s_at | -1.4 | 4.1.E-04 | Fraser syndrome 1 | FRAS1 | chr4q21.21 | calcium ion binding |
| 226380_at | -1.1 | 2.4.E-04 | protein tyrosine phosphatase, non-receptor type 21 | PTPN21 | chr14q31.3 | phosphoprotein phosphatase activity |
| 226462_at | -1.8 | 5.1.E-02 | syntaxin binding protein 6 (amisyn) | STXBP6 | chr14q12 | --- |
| 226757_at | -1.2 | 7.3.E-04 | interferon-induced protein with tetratricopeptide repeats 2 | IFIT2 | chr10q23-q25 | binding |
| 226769_at | -1.4 | 5.7.E-04 | fin bud initiation factor homolog | FIBIN | chr11p14.2 | --- |
| 226950_at | -1.7 | 4.1.E-04 | activin A receptor type II-like 1 | ACVRL1 | chr12q11-q14 | nucleotide binding |
| 227197_at | -1.5 | 2.4.E-04 | Src homology 3 domain-containing guanine nucleotide exchange factor | SGEF | chr3q25.2 | guanyl-nucleotide exchange factor activity |
| 227198_at | -1.5 | 2.4.E-04 | AF4/FMR2 family, member 3 | AFF3 | chr2q11.2-q12 | DNA binding |
| 227848_at | -1.4 | 2.4.E-04 | phosphatidylethanolamine-binding protein 4 | PEBP4 | chr8p21.3 | --- |
| 228184_at | -1.5 | 2.4.E-04 | dispatched homolog 1 | DISP1 | chr1q41 | hedgehog receptor activity |
| 228434_at | -1.9 | 4.7.E-03 | butyrophilin-like 9 | BTNL9 | chr5q35.3 | --- |
| 228977_at | -1.5 | 1.5.E-03 | hypothetical protein LOC729680 | LOC729680 | chr13q12.11 | --- |
| 229125_at | -2.1 | 1.9.E-02 | KN motif and ankyrin repeat domains 4 | KANK4 | chr1p31.3 | --- |
| 229292_at | -1.2 | 3.0.E-03 | erythrocyte membrane protein band 4.1 like 5 | EPB41L5 | chr2q14.2 | binding |
| 229309_at | -1.7 | 2.4.E-04 | adrenergic, beta-1-, receptor | ADRB1 | chr10q24-q26 | signal transducer activity |
| 229450_at | -1.3 | 2.4.E-04 | interferon-induced protein with tetratricopeptide repeats 3 | IFIT3 | chr10q24 | binding |
| 229985_at | -2.0 | 5.7.E-04 | Butyrophilin-like 9 | BTNL9 | chr5q35.3 | --- |
| 230135_at | -1.7 | 7.3.E-04 | --- | --- | --- | --- |
| 230250_at | -1.4 | 2.4.E-04 | protein tyrosine phosphatase, receptor type, B | PTPRB | chr12q15-q21 | phosphoprotein phosphatase activity |
| 230360_at | -1.8 | 2.4.E-04 | gliomedin | GLDN | chr15q21.2 | --- |
| 230469_at | -3.3 | 4.1.E-04 | rhotekin 2 | RTKN2 | chr10q21.2 | --- |
| 230560_at | -2.3 | 2.4.E-04 | syntaxin binding protein 6 | STXBP6 | chr14q12 | --- |
| 230959_at | -1.3 | 2.4.E-04 | --- | --- | --- | --- |
| 231804_at | -1.5 | 9.8.E-04 | relaxin/insulin-like family peptide receptor 1 | RXFP1 | chr4q32.1 | signal transducer activity |
| 231925_at | -1.5 | 1.4.E-03 | --- | --- | --- | --- |
| 232080_at | -1.7 | 2.4.E-04 | HECT, C2 and WW domain containing E3 ubiquitin protein ligase 2 | HECW2 | chr2q32.3-q33.1 | protein binding |
| 232578_at | -2.4 | 4.1.E-04 | claudin 18 | CLDN18 | chr3q22.3 | structural molecule activity |
| 233903_s_at | -1.4 | 4.1.E-04 | Src homology 3 domain-containing guanine nucleotide exchange factor | SGEF | chr3q25.2 | guanyl-nucleotide exchange factor activity |
| 234996_at | -1.5 | 2.4.E-04 | calcitonin receptor-like | CALCRL | chr2q32.1 | calcitonin gene-related polypeptide receptor activity |
| 235228_at | -1.8 | 1.1.E-03 | coiled-coil domain containing 85A | CCDC85A | chr2p16.1 | --- |
| 235568_at | -2.3 | 2.4.E-04 | chromosome 19 open reading frame 59 | C19orf59 | chr19p13.2 | --- |
| 235591_at | -2.2 | 1.5.E-03 | somatostatin receptor 1 | SSTR1 | chr14q13 | signal transducer activity |
| 236089_at | -1.9 | 5.7.E-04 | --- | --- | --- | --- |
| 236313_at | -1.6 | 2.4.E-04 | cyclin-dependent kinase inhibitor 2B (p15, inhibits CDK4) | CDKN2B | chr9p21 | cyclin-dependent protein kinase inhibitor activity |
| 236383_at | -1.2 | 2.8.E-03 | --- | --- | --- | --- |
| 236712_at | -2.1 | 7.3.E-04 | --- | --- | --- | --- |
| 237466_s_at | -1.8 | 1.1.E-03 | hedgehog interacting protein | HHIP | chr4q28-q32 | catalytic activity |
| 238206_at | -1.9 | 1.1.E-02 | relaxin/insulin-like family peptide receptor 1 | RXFP1 | chr4q32.1 | signal transducer activity |
| 239650_at | -2.3 | 2.6.E-03 | NCK-associated protein 5 | NCKAP5 | chr2q21.2 | protein binding |
| 241672_at | -2.1 | 7.3.E-04 | chromosome 13 open reading frame 36 | C13orf36 | chr13q13.3 | --- |
| 241811_x_at | -3.5 | 6.5.E-03 | solute carrier family 6 (neurotransmitter transporter, serotonin), member 4 | SLC6A4 | chr17q11.1-q12 | neurotransmitter:sodium symporter activity |
| 242009_at | -5.6 | 2.4.E-04 | solute carrier family 6 (neurotransmitter transporter, serotonin), member 4 | SLC6A4 | chr17q11.1-q12 | neurotransmitter:sodium symporter activity |
| 242332_at | -1.2 | 3.0.E-03 | hypothetical LOC283904 | LOC283904 | chr16q24.1 | --- |
| 243813_at | -2.2 | 5.7.E-04 | --- | --- | --- | --- |
| 243929_at | -1.8 | 2.4.E-04 | --- | --- | --- | --- |
| 1552701_a_at | -1.1 | 2.4.E-04 | caspase recruitment domain family, member 16 | CARD16 | --- | cysteine-type endopeptidase activity |
| 1552715_a_at | -2.1 | 2.0.E-03 | relaxin/insulin-like family peptide receptor 1 | RXFP1 | chr4q32.1 | signal transducer activity |
| 1556037_s_at | -1.9 | 1.1.E-03 | hedgehog interacting protein | HHIP | chr4q28-q32 | catalytic activity |
| 1558444_at | -1.4 | 2.4.E-04 | --- | --- | --- | --- |
| 1560850_at | -2.3 | 5.7.E-04 | --- | --- | --- | --- |

^*^ Probe name in Affymetrix.

^†^ SLR = signal log ratio. The SLR algorithm measures the magnitude and direction of the change between signal levels of the emphysematous lesions versus fibrotic lesions

^‡^ The significance of the signal changes between the emphysematous lesions versus fibrotic lesions. For example, P=7.3.E-04 indicates P= 7.3 x 10^-4^.

--- Unknown or undetected.
